# Puerarin improves diabetic wound healing via regulation of macrophage M2 polarization phenotype

**DOI:** 10.1093/burnst/tkac046

**Published:** 2022-12-22

**Authors:** Shiyan Li, Ping Yang, Xiaofeng Ding, Hao Zhang, Youjun Ding, Qian Tan

**Affiliations:** Department of Burns and Plastic Surgery, Nanjing Drum Tower Hospital, the Affiliated Hospital of Nanjing University Medical School, NO. 321, Zhongshan Road, Nanjing, Jiangsu, China; Department of Burns and Plastic Surgery, Nanjing Drum Tower Hospital, the Affiliated Hospital of Nanjing University Medical School, NO. 321, Zhongshan Road, Nanjing, Jiangsu, China; Department of Burns and Plastic Surgery, Nanjing Drum Tower Hospital Clinical College of Traditional Chinese and Western Medicine, Nanjing University of Chinese Medicine, NO. 321, Zhongshan Road, Nanjing, Jiangsu 210008, China; Department of Burns and Plastic Surgery, Nanjing Drum Tower Hospital, the Affiliated Hospital of Nanjing University Medical School, NO. 321, Zhongshan Road, Nanjing, Jiangsu, China; Department of Burns and Plastic Surgery, Nanjing Drum Tower Hospital Clinical College of Jiangsu University, NO. 321, Zhongshan Road, Nanjing, Jiangsu 210008, China; Department of Burns and Plastic Surgery, Nanjing Drum Tower Hospital, the Affiliated Hospital of Nanjing University Medical School, NO. 321, Zhongshan Road, Nanjing, Jiangsu, China; Department of Burns and Plastic Surgery, Anqing Shihua Hospital, Nanjing Drum Tower Hospital Group, Anqing 246002, China

**Keywords:** Macrophage, Puerarin, Wound healing, Diabetes, Skin, Streptozotocin, Traditional Chinese medicine, Nuclear factor kinase B, Mitogen-activated protein kinase

## Abstract

**Background:**

Skin wound healing depends on the progress of different but overlapping stages of healing, including hemostasis, inflammatory, proliferative and remodeling. Failure of these stages to occur in a timely and gradual manner may result in non-healing pathological wounds. Macrophages and neutrophils have been shown to play an essential role in the inflammatory responses of wound tissue, and their active plasticity allows them to modulate tissue damage and repair functions. The ability of macrophages and neutrophils to regulate the occurrence and resolution of inflammatory processes is essential for the treatment of pathological wound healing.

**Methods:**

Mice were categorized into negative control, streptozotocin, streptozotocin + puerarin and puerarin groups. The traditional Chinese medicine extract puerarin was selected to treat different groups of mice with a full-thickness skin defect wound. Cells of the RAW264.7 cell line were stimulated under different puerarin conditions. Then, real time quantitative polymerase chain reaction (RT-qPCR), western blot, immunofluorescence and other assays were carried out to explore the effect of puerarin on wound healing and its molecular mechanism.

**Results:**

Animal experiments found that the wound healing of diabetic mice treated with puerarin was significantly accelerated, and histological analysis found that puerarin treatment markedly decreased the infiltration of macrophages and neutrophils in wound tissue. Through western blot, RT-qPCR and immunofluorescence experiments, it was observed that puerarin treatment remarkably inhibited nuclear factor kinase B (NF-κB) and mitogen-activated protein kinase (MAPK) signaling pathways, downregulated the expression of inflammatory cytokines and induced the M2 polarization of macrophages. At the cellular level, we also observed that puerarin improved M2 macrophage polarization and inhibited inflammatory pathway activation in a high-glucose culture.

**Conclusion:**

Puerarin has a significant therapeutic effect on wound healing in diabetic mice. The therapeutic effect is achieved by regulating macrophage polarization through suppressing NF-κB and MAPK signaling cascades.

Highlights

- Intraperitoneal injection of puerarin can promote wound healing in diabetic mice.
- Puerarin treatment remarkably induced the M2 polarization of macrophages in wound tissue of diabetic mice.
- Puerarin improved M2 macrophage polarization in a high-glucose culture medium.
- The effects of puerarin on macrophage polarization are probably related to NF-κB and MAPK signaling pathways.

## Background

Cutaneous wound healing is a complex process comprised of several steps and molecular mechanisms, which requires a number of organic, epigenetic and other biological layers to achieve [1,2]. Skin tissue repair is highly regulated under normal conditions, but a defect in this process can disturb the subtle balance of gene products and signaling cascades in cells, thus affecting the process of wound healing [3,4]. There are four overlapping stages of hemostasis, inflammatory, proliferative and remodeling, which require the orchestrated integration of molecular and cellular events as well as signaling pathways [5]. However, in diabetic wounds the four stages are dysregulated, and an excessive inflammatory response can lead to a difficult transition from inflammation to remodeling [6,7]. Therefore, how to suppress excessive inflammation of diabetic wounds is a meaningful problem and awaits further investigation.

Excessive inflammation of diabetic wounds is associated with macrophages and neutrophils. In the inflammatory phase, the plasticity of macrophages is of great importance for wound tissue healing and remodeling [8]. Macrophages and neutrophils induce an inflammatory response and tissue damage at an early stage of inflammation, and the modulation of these processes is beneficial to wound healing. In the late inflammatory phase, macrophages differentiate into the M2 type and move into the proliferative phase [9]. Manifestations of early inflammation are increased inflammation cytokines and pathogen killing ability, while macrophages in late inflammation phases yield anti-inflammatory cytokines during the transition from the inflammatory stage to the proliferative stage [10,11]. These phenotypic macrophages dominate at specific time points during wound healing and contribute to the formation of customized macrophage-dependent responses. Macrophage polarization is crucial for the process of diabetic wound healing. Macrophages fall into two main categories, classically activated macrophages (M1) and selectively activated macrophages (M2) [12]. M1 macrophages produce pro-inflammatory factors, which can contribute to organ dysfunction. On the other hand, M2 macrophages release anti-inflammatory mediators that attenuate inflammation [13,14]. It is worth noting that improving M2 properties and decreasing M1 characteristics may help diabetic wound healing [15].

Many studies have exhaustively assessed the regulation of macrophages in response to inflammatory stimuli in cells and animal models. Lipopolysaccharide (LPS) mediates pro-inflammatory induction by activating the main transcription factor nuclear factor κB (NF-κB), which leads to M1 polarization [16]. It is well known that RelA protein mediates the typical NF-κB pathway, and its destruction can downregulate the effects of NF-κB such as the formation of pro-inflammatory mediators [17]. Under normal physiological conditions, NF-κB protein is isolated in the cytoplasm, whereas under certain stimulation, this protein can form homodimers or heterodimers and interact with inhibitors. Inhibitor of NF-κB (IκB) protein is translocated into the nucleus where it mediates the expression of downstream target genes of NF-κB [18]. Apart from NF-κB and its downstream signals, the ubiquitous redox proteins and cytokine microenvironment can also regulate other proteins, including signal transducers and activators of transcription (STATs) [19]. These members are regulated by different stimuli [e.g. interleukin-6 (IL-6)], and subsequently activate the expression of STAT3 and IL-4 that regulate the downstream effectors (e.g. STAT6) [20]. In the complex environment of cell signaling molecules, STAT protein regulation of macrophage polarization has been observed. Emerging research revealed epigenetic changes in the regulation of STAT6 via upstream mediators [21]. The involvement of macrophages, from induction and wound healing to tumor monitoring and inhibition, may have far-reaching consequences. Among all STAT subtypes, the functions of STAT3 in macrophage polarization have attracted great attention because of its contradictory roles in affecting the polarization results [22]. In the presence of IL-6, M2 polarizes LPS-exposed bone marrow-derived macrophages to secrete IL-10 through STAT3, thereby reducing the production of cytokines [23]. Recent evidence suggests that JSI-124 (a STAT3 inhibitor) transiently upregulates the NF-κB pathway *in vitro* and stimulates human glioblastoma cell apoptosis [24].

Puerarin is an isoflavonoid extracted from *Pueraria lobata* roots, and has been used for various medicinal purposes in traditional oriental medicine. According to previous studies, puerarin is known to have therapeutic effects in angiocardiopathy, osteoporosis, liver damage, cancers and diabetes [25]. Several studies have substantiated that puerarin treatment induced a marked improvement in insulin resistance and defective β-cell secretions [26,27]. The potential hypoglycemic mechanism of puerarin is related to activation of the phosphatidylinositol 3-kinase (PI3K)/protein kinase B (Akt) signaling pathway and inhibition of reactive oxygen species (ROS) production in the pancreas [28]. However, the effect of puerarin on diabetic wound repair has not been determined. Therefore, this research aimed to explore whether puerarin treatment can induce M2 polarization, attenuate inflammation and promote wound healing in diabetic mice.

## Methods

### Antibodies and reagents

PE cluster of differentiation 206 (CD206) antibody (162503) and FITC mouse EGF-like module-containing mucin-like hormone receptor-like 1 (F4/80) antibody (157309) were procured from Biolegend. Arginase 1 (Arg-1; 93 668), lymphocyte antigen 6 complex (Ly6G) (68590), F4/80 (70076), CD206 (24595), p-P65 (phospho-nuclear factor kappa B) (3033), p38 MAP kinase (p38) (8690) and phosphorylated STAT3 (p-STAT3) (9145) were purchased from CST. Phospho-extracellular signal-related kinase (p-ERK) (ab201015), glyceraldehyde 3-phosphate dehydrogenase (GAPDH) (ab181602), inducible nitric oxide synthase (iNOS) (ab178945), cytokeratin 5 (K5) (ab52635), tumor necrosis factor-α (TNF-α) (ab183218), IL-6 (ab290735) and IL-1β (ab254360) were purchased from Abcam. P65 (A19653), phospho-nuclear factor of kappa alpha (p-IkBα) (AP0707), phospho-p38 (p-p38) (AP0057), phospho-c-Jun N-terminal kinase (p-JNK) (AP0631), JNK (A4867) and ERK (A4782) were purchased from ABclonal. Puerarin (P5555) was supplied by Merck. Trizol Reagent and SYBR green were purchased from Vazyme Biotech. Streptozotocin (S0130) and glucose (D9434) were obtained from Sigma-Aldrich.

### Experimental animals

The experiment was conducted according to the local guidelines and approved by Nanjing Drum Tower Hospital Animal Care and Use Committee. Male C57BL/6 mice (aged 6–8 weeks, 20–25 g) were provided by the Model Animal Research Center of Nanjing University. These mice were housed under normal laboratory conditions (a controlled temperature of 25°C and circadian light/dark cycles) in accordance with the specific pathogen-free standard, with unlimited access to water and food.

### Establishment of diabetic mouse models and treatment *in vivo*

All mice were divided into four groups: negative control (NC), streptozotocin (STZ), streptozotocin + puerarin (STZ + PUE) and puerarin (PUE), with 18 mice in each group. A full-thickness wound was excised on the back of the mice after diabetes induction. The mice in STZ and STZ + PUE groups were injected daily with STZ (50 mg/kg intraperitoneal injection [i.p.]; Sigma, USA) for 5 days. After 1 week, a glucometer was used to examine hyperglycemia. Only mice with blood glucose (BG) level > 300 mg/dl were significantly different from control mice and were subjected to further analysis. Once the diabetes mouse model was established, PUE (120 mg/kg i.p.) was administered daily to the mice. Meanwhile, the control mice received the same volume injection of phosphate buffered saline (PBS). The non-diabetic mice were also treated with PUE alone to evaluate the related indicators. On days 5 and 10, wound areas were sampled for hematoxylin and eosin (H&E), Masson’s trichrome (MT), immunohistochemistry, immunofluorescence, real time quantitative polymerase chain reaction (RT-qPCR) and western blot analyses.

### Wound model construction

Mice were anesthetized by injecting 0.6% sodium pentobarbital (10 ml/kg i.p.). A chemical depilatory was used to remove the back hair, followed by the development of full-thickness skin wounds using a punch biopsy (diameter = 8 mm). Measurement of wound size was conducted postoperatively every other day. After anesthetization with isoflurane, the wound images were captured. ImageJ was used to analyze wound areas. All mice were euthanized after their last treatment, and wound tissues were isolated and preserved in paraformaldehyde (4%) overnight. Subsequently, the specimens were paraffin-embedded and subjected to histochemical staining.

### Cell culture and treatment procedures

RAW264.7 cells were provided by the Chinese Academy of Sciences cell bank and then cultured in Dulbecco's modified Eagle's medium (DMEM) containing 1% penicillin–streptomycin and 10% fetal bovine serum (FBS) (GIBCO, NY, USA). The cells were primarily categorized into four groups: NC (containing 5.5 mM D-glucose), HG (high glucose; containing 50 mM D-glucose), HG + PUE (containing 120 μΜ puerarin and 50 mM D-glucose) and NC + PUE (containing 120 μΜ puerarin and 5.5 mM D-glucose). In order to mimic the inflammatory environment of a diabetic wound, LPS (100 ng/ml) was added for 24 h before cell treatment. After rehydrating RAW264.7 cells onto 6-well plates, they were maintained in an incubator at 37°C and 5% CO_2_. In the cell counting kit-8 (CCK8) test, 1 × 10 ^4^ cells per well were cultured in a 96-well plate and incubated with CCK8 reagent for 2 h.

### H&E and MT staining

The wounds with surrounding tissues were fixed in paraformaldehyde (4%) and then subjected to standard histological procedures and fixed embedding. Microtomy was performed to prepare tissue sections of 5 μm thickness, followed by H&E and MT staining. In addition, the histological wound healing score was computed by determining indicators such as re-epithelization, scar elevation index, granulation tissue thickness and remodeling in each mouse [29].

**Table 1:** Sequences of the primer pairs employed for RT-qPCR

**Gene**	**Source**	**Forward**	**Reverse**
F4/80	Mouse	TGACTCACCTTGTGGTCCTAA	CTTCCCAGAATCCAGTCTTTCC
Cd11b	Mouse	GGGAGGACAAAAACTGCCTCA	ACAACTAGGATCTTCGCAGCAT
TNF-α	Mouse	GACGTGGAACTGGCAGAAGAG	GCCACAAGCAGGAATGAGAAG
IL-1β	Mouse	CTTCCCCAGGGCATGTTAAG	ACCCTGAGCGACCTGTCTTG
Arg1	Mouse	CTCCAAGCCAAAGTCCTTAGAG	AGGAGCTGTCATTAGGGACATC
IL-10	Mouse	GGTTGCCAAGCCTTATCGGA	ACCTGCTCCACTGCCTTGCT
CD206	Mouse	CTCTGTTCAGCTATTGGACGC	CGGAATTTCTGGGATTCAGCTTC
CD163	Mouse	ATGGGTGGACACAGAATGGTT	CAGGAGCGTTAGTGACAGCAG
TGF-β1	Mouse	CTTCAATACGTCAGACATTCGGG	GTAACGCCAGGAATTGTTGCTA
GAPDH	Mouse	GCACCGTCAAGGCTGAGAAC	TGGTGAAGACGCCAGTGGA

*TNF-α *tumor necrosis factor-α, *IL-1*β interleukin-1β, *Arg1* arginase 1, *CD206* cluster of differentiation 206, *CD163* cluster of differentiation 163, *TGF-β1* transforming growth factor beta 1, *GAPDH* glyceraldehyde 3-phosphate dehydrogenase *RT-qPCR* real time quantitative polymerase chain reaction

### Immunohistochemistry

The wound tissues of mice were fixed in paraformaldehyde (4%) and then embedded in paraffin. Serial longitudinal 5-μm thick sections were cut, deparaffinized and rehydrated. Each section was blocked by incubation with normal bovine serum albumin (BSA) serum at room temperature (RT) for 1 h and then incubated in Ly6G, F4/80 and p-STAT3 antibodies (1:100) at 4°C. The sections were washed with PBS, followed by a 2-h exposure to biotinylated secondary antibodies at RT.

### Immunofluorescence analysis

To evaluate the intracellular immune infiltration of neutrophils and macrophages, wound tissue was cultured on a sterile cover. After a specified treatment, the tissue was fixed in 10% formaldehyde in PBS for 10 min, and then rinsed three times with PBS. The tissue was then blocked in 5% normal serum–0.25% Triton X-100 in PBS for 1 h at RT. Next, incubation was conducted with anti Ly6G, CD206, iNOS, F4/80 or K5 (1:200) at 4°C overnight. After rinsing with PBS three times for 5 min each, the tissue was exposed to Alexa Fluor 488-labeled goat anti-rabbit antibody (1:1000) for 20 min. 4′,6-diamidino-2-phenylindole (DAPI) was used to stain the nucleus, followed by confocal scanning microscopy (Leica TCS-SP2, Heidelberg, Germany). The fluorescence intensities of Ly6G and F4/80 in the center were analyzed with Image-Pro Plus v6.0 software.

RAW264.7 cells were grown in 24-well plates and then subjected to the indicated treatments for 48 h. After fixing in paraformaldehyde (4%) for 30 min, the cells were rinsed three times with PBS for 5 min each, and then exposed to 0.1% Triton-X-100 in phosphate buffered saline tween (PBST) for 15 min at RT. After incubation with 5% BSA for 1 h, the primary antibody was incubated overnight at 4°C. After rinsing three times with PBST for 5 min each, the cells were exposed to fluorescent secondary antibody at RT for 1 h in the dark. DAPI staining was performed for 3 min in the dark, followed by rinsing with PBST three times for 5 min each. After sealing, the stained cells were examined by laser scanning confocal microscopy.

### RT-qPCR

Total RNA of the mouse wound tissue and RAW264.7 cell line was extracted using the Vazyme biotech reagent (Vazyme biotech, China). A 1 μg amount of the total RNA was subjected to cDNA synthesis via a reverse transcription kit. GAPDH was employed as a housekeeping gene. Table 1 lists the primer sequences of each target gene. Power SYBR Green PCR Master Mix was used to perform RT-qPCR on an ABI Viia 7 detector system according to the standard procedure. The relative mRNA expression of target genes was evaluated by the ΔCT method, and the formula was as follows: 2^−ΔΔCt^.

### Western blotting

The mouse wound tissue and RAW264.7 cells were lysed with a Total Protein Extraction Kit (Beyotime, China) and total protein content was determined with the BCA protein assay (Beyotime, China). After separating through 10% sodium dodecyl sulfate-polyacrylamide gel electrophoresis (SDS-PAGE), the protein samples (50 μg) were transferred onto a polyvinylidene fluoride (PDVF) membrane (BioRad, USA). The membrane was blocked with 5% non-fat milk in tris buffered saline tween (TBST) and then incubated with the following primary antibodies: GAPDH, CD206, Arg-1, TNF-α, IL-6, IL-1β, p65, p-IkBα, p-p38, p-JNK and p-ERK. Incubation with horseradish peroxidase (HRP)-labeled anti-goat or anti-rabbit secondary antibodies was then executed. Lastly, the protein blots were visualized with ECL reagent (Vazyme, China) and then recorded using a chemiluminescent imaging system (Tanon, China).

### Myeloperoxidase (MPO) activity assay and enzyme-linked immunosorbent assay (ELISA)

MPO is produced by neutrophils and serves as a biomarker of inflammation. After excision and homogenization at 4°C, the protein concentration of each specimen was calculated using the BCA protein assay kit. MPO activity was evaluated by a commercial kit (Nanjing Jiancheng Biotech Co. Ltd, China) using a chromatometer according to the kit’s protocols, and the results of MPO activity are shown as U/g of protein. Blood samples were taken from the eyes of each mouse. Meanwhile, the levels of IL-6, IL-10 and TNF-α were measured with ELISA kits (BYabscience Biotechnology, China) by following the kit’s instructions.

### Flow cytometry

RAW264.7 cells were prepared as a single-cell suspension. Fluorescein isothiocyanate (FITC)-labeled anti-mouse F4/80 monoclonal antibody and P-phycoerythrin (PE)-labeled anti-mouse CD206 monoclonal antibody were used to assess the polarization trend of macrophages, and were stained for 30 min in the dark. Staining of CD206 was conducted through perforations in the cytomembrane. Flow cytometry was executed using a FACS flow cytometer (BD Biosciences). FlowJo software was utilized for data analysis.

### Statistical analysis

Experimental data were analyzed with Graphpad Prism v8.0 software and are presented as mean ± SD. Parametric tests were used for data that conform to the normal distribution (Shapiro–Wilk test). If the data were normally distributed, the statistical differences among multiple groups were compared by one-way analysis of variance (ANOVA) and Newman–Keuls *post hoc* test. The two tailed Student’s t test was applied for the comparison of two groups, if the data passed the normality test. The combination effects of two factors were analyzed with two-way ANOVA followed by Tukey’s post-test. At least three independent assays were conducted, and *p*-values of <0.05 were deemed statistically significant.

**Figure 1.**
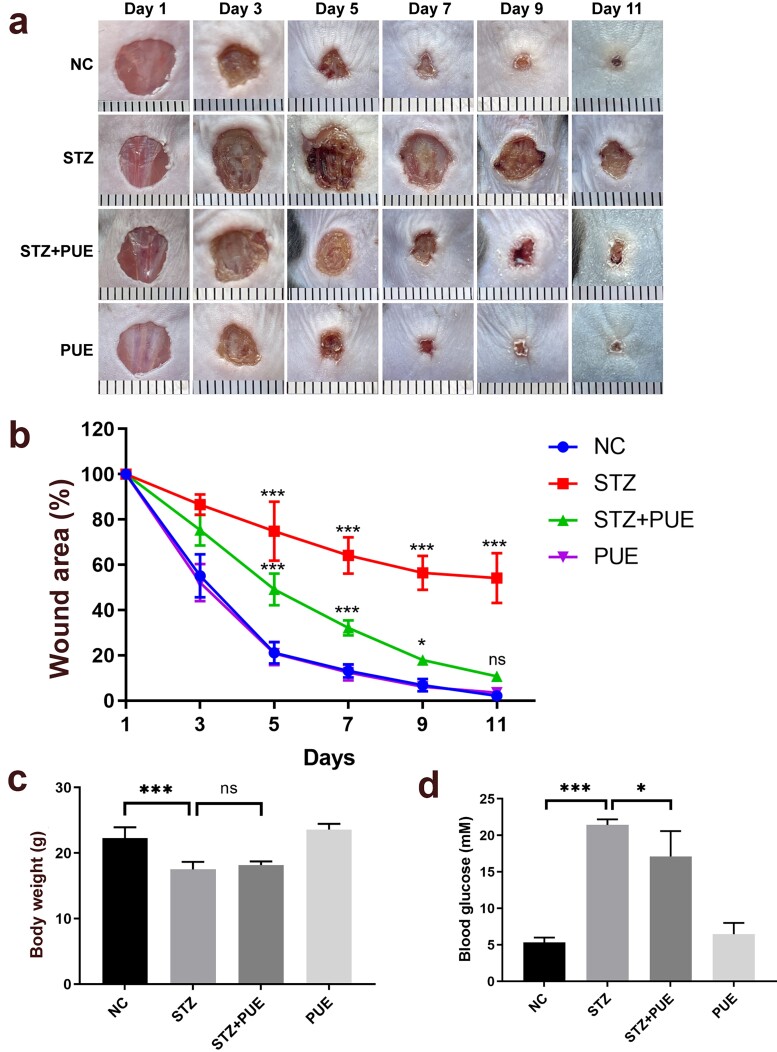
Treatment with puerarin ameliorates wound healing in STZ-induced diabetic mice. (**a**) Data for wound changes were recorded daily from day 1 to day 11. (**b**) Wound area data were measured every 2 days from 1 to 11 days; (**c**, **d**) Measured body weight and blood glucose of the experimental mice. *n* = 6, ^*^p < 0.05, ^***^p < 0.001. *ns* no statistical significance, *NC* negative control, *STZ* streptozotocin, *PUE* puerarin

**Figure 2.**
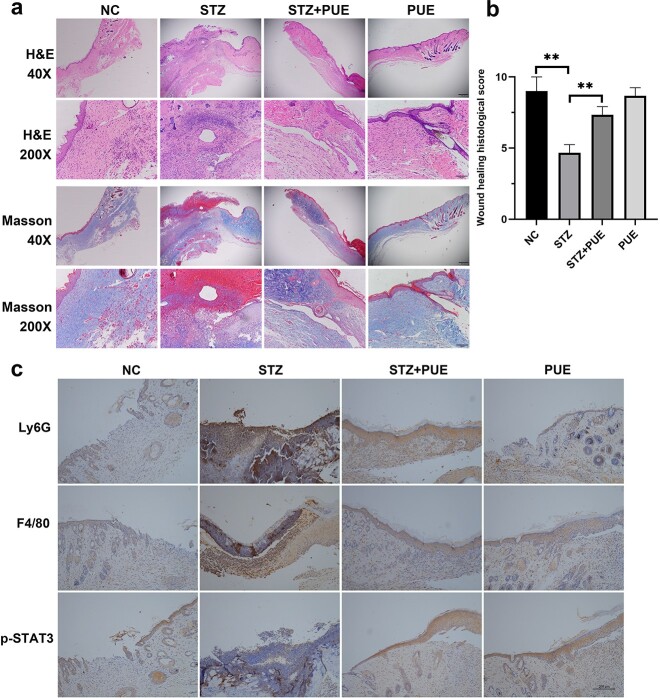
Effect of PUE treatment on the histological and immunohistochemical changes in wound tissues. (**a**) H&E and Masson staining of representative wounds in control, STZ, STZ + PUE and PUE groups on day 10 post-injury (Scale bar: 50μm, Scale bar: 200μm). (**b**) Quantitative analysis of the wound healing histological score. (**c**) Expression levels of Ly6G, F4/80 and p-STAT3 were examined by immunohistochemistry on day 10 post-injury (Scale bar: 200 µm) . *n* = 6, **p < 0.01. *NC* Negative control, *STZ* streptozotocin, *PUE* puerarin, *H&E* hematoxylin and eosin staining

**Figure 3.**
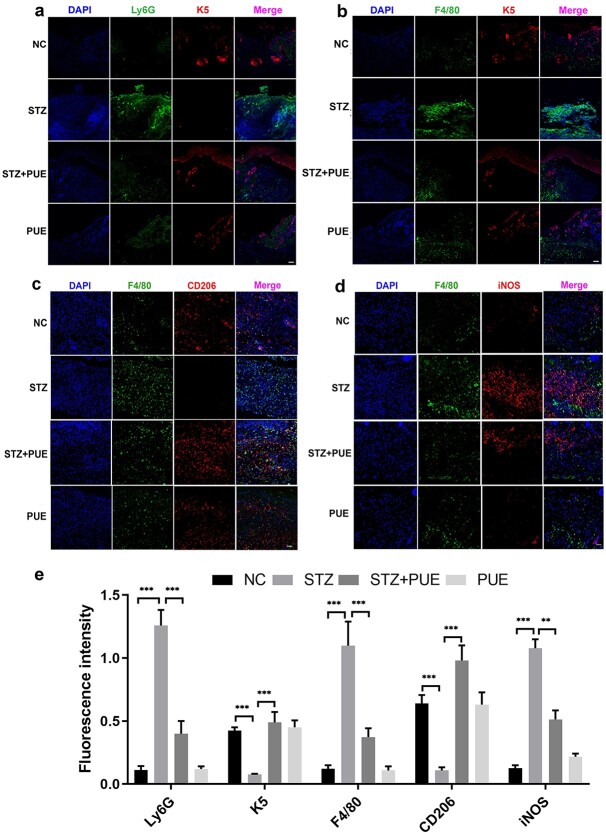
Immunofluorescence analysis reveals the effect of puerarin on neutrophils and macrophages. (**a**, **b**) Effect of PUE on infiltrated immunocytes was determined via immunofluorescence on day 10 post-injury. Red fluorescence represents cytokeratin 5 (K5); green fluorescence represents Ly6G and F4/80; blue fluorescence represents cell nucleus. (**c**, **d**) Immunofluorescence analysis of CD206+ (red), iNOS+ (red) and F4/80+ (green) cells in NC, STZ, STZ + PUE and PUE on day 10 post-injury. (**e**) Quantified fluorescence intensity of Ly6G, K5, F4/80, CD206, iNOS. *n* = 6, ^**^p < 0.01, ^***^p < 0.001. Scale bar: 100 μm. *NC* negative control, *STZ* streptozotocin, *PUE* puerarin, *DAPI* 4′6-diamidino-2-phenylindole, *CD206* cluster of differentiation 206, *iNOS* inducible nitric oxide synthase

**Figure 4.**
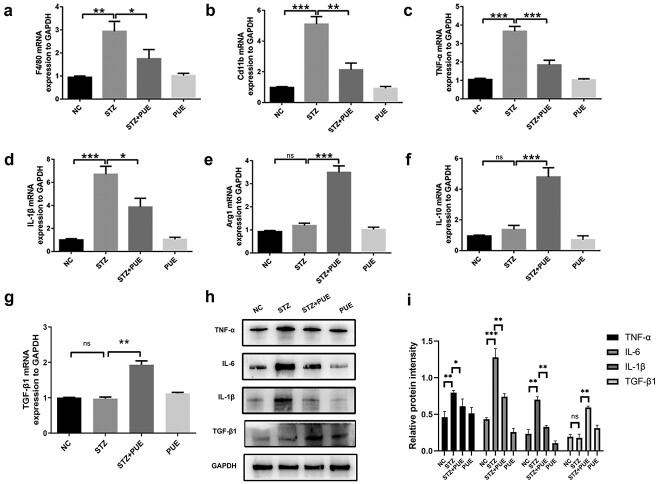
Puerarin decreases the infiltrated immunocytes of neutrophils and macrophages in wound tissue, as revealed by RT-qPCR and western blot analyses. (**a**–**g**) Expression levels of F4/80, Cd11b, TNF-α, IL-1β, Arg-1, IL-10 and TGF-β1 were detected by RT-qPCR. (**h**) TNF-α, IL-6, IL-1β and TGF-β1 were detected by Western blot and GAPDH was used as an internal reference. (**i**) Western blot bands shown in (h) were analyzed by densitometry. *n* = 6, ^*^p < 0.05, ^**^p < 0.01, ^***^p < 0.001. *ns* no statistical significance, *NC* negative control, *PUE* puerarin *TNF-α* tumor necrosis factor-α, *TGF-β1* transforming growth factor beta1, *IL-1β* interleukin-1β, Arg1 arginase 1, *IL-10* interleukin-10, *IL-6* interleukin-6, *GAPDH* glyceraldehyde 3-phosphate dehydrogenase

**Figure 5.**
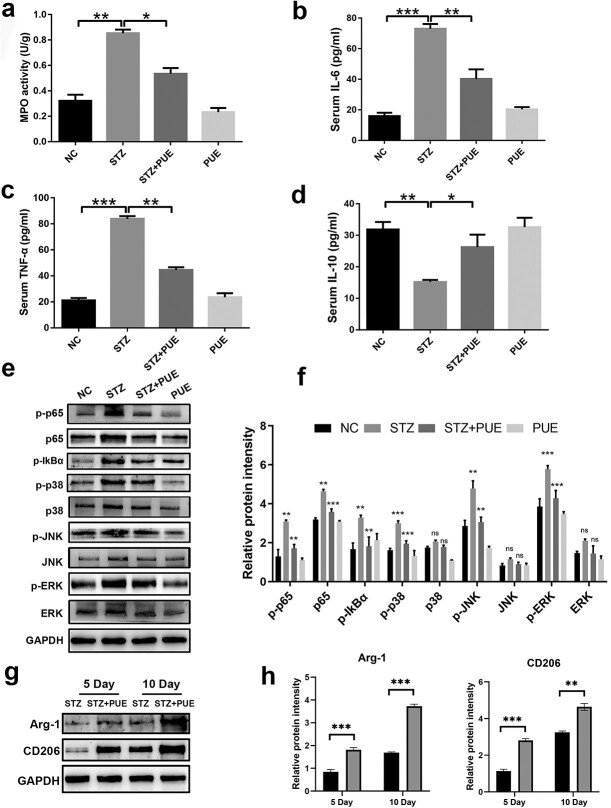
Puerarin inhibits inflammation by inhibiting NF-κB and MAPK signaling cascades in the wound tissue of diabetic mice. (**a**–**d**) MPO activity as well as serum IL-6, TNF-α and IL-10 levels were detected. (**e**, **f**) Western blotting was conducted to detect the protein levels of p-p65, p65, p-IkBα, p-p38, p38, p-JNK, JNK, p-ERK, ERK and GAPDH in mice. The relative protein intensity was normalized to GAPDH. (**g**, **h**) The protein levels of CD206 and Arg-1 in the wound tissues of STZ and STZ + PUE groups on days 5 and 10 were evaluated by Western blotting. The relative protein intensity was normalized to GAPDH. *n* = 6, ^*^p < 0.05, ^**^p < 0.01, ^***^p < 0.001. *ns *no statistical significance, *NC* negative control, *STZ* streptozotocin, *PUE* puerarin, *MPO* myeloperoxidase, *IL-10* interleukin-10, *IL-6* interleukin-6, *TNF-α* tumor necrosis factor-α, *GAPDH* glyceraldehyde 3-phosphate dehydrogenase

## Results

### Puerarin improves wound healing in diabetic mice

Male C57BL/6 mice (aged 6–8 weeks, 20–25 g) were were used for experiments. To assess the efficacy of puerarin on wound healing in diabetic mice, full-thickness back wound areas were recorded on days 1, 3, 5, 7, 9 and 11 (Figure 1a, b). Compared to the STZ group, the size of the wound in the STZ + PUE group was reduced significantly on day 5. In the same way, the trends from day 7 to day 11 appeared to be similar. In addition, we also observed the weight and BG measurements of mice on day 11 (Figure 1c, d). Notably, the body weight (BW) of the STZ group was relatively similar to that of the STZ + PUE group after 11 days of PUE treatment, and the BWs of the STZ and STZ + PUE groups decreased significantly compared to the NC group. Meanwhile, after 11 days of PUE treatment, the BG of the STZ + PUE mice decreased slightly compared with that of the STZ mice. Based on these results, puerarin appears to have therapeutic effects on the diabetic mice induced by STZ.

**Figure 6.**
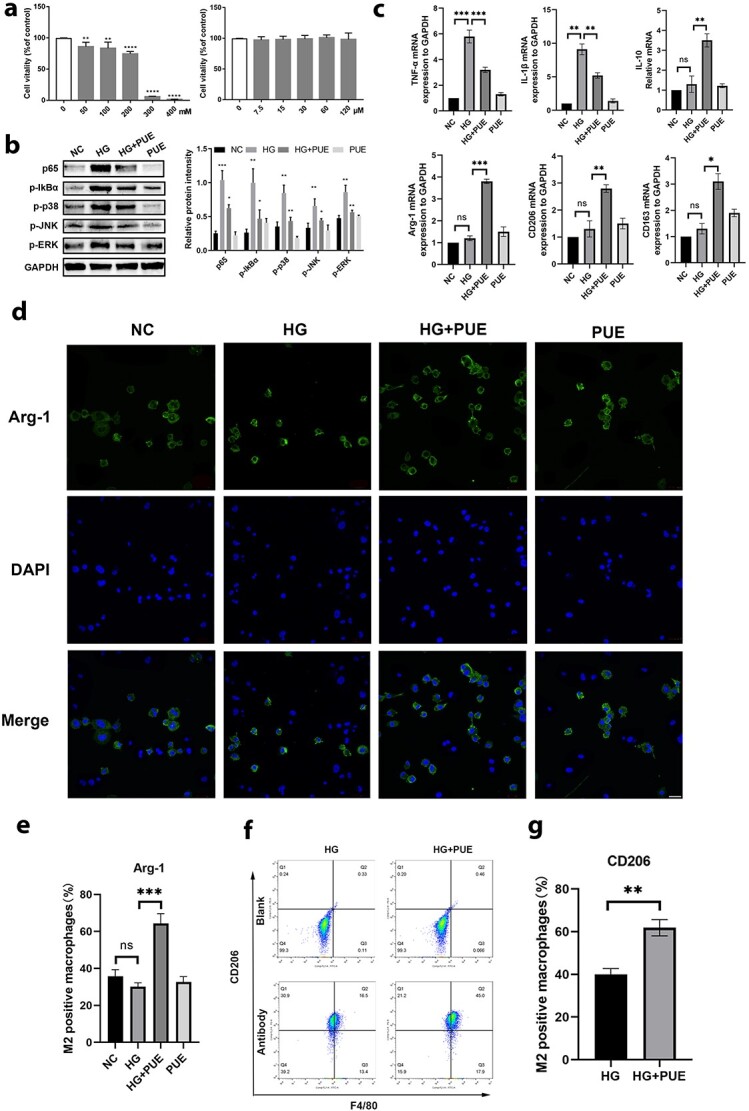
Puerarin can inhibit inflammation and induce M2 polarization *in vitro* in a high-glucose environment. (**a**) CCK8 experiment showed the cytotoxicity of puerarin in a high-glucose culture. (**b**) Western blot assay was conducted to assess the expression levels of p65, p-IkBα, p-p38, p-JNK, p-ERK and GAPDH proteins in RAW 264.7 macrophages. The relative protein intensity was normalized to GAPDH. (**c**) mRNA abundance of TNF-α, IL-1β, IL-10, Arg-1, CD206 and CD163 detected by RT-qPCR. (**d**, **e**) Immunofluorescence staining of NC, HG, HG + PUE and PUE groups revealed Arg-1+ cells. (**f**, **g**) Flow cytometry showed the difference in the quantities of M2 macrophages between HG and HG + PUE groups. Blank: no flow cytometry antibody. Antibody: antibody incubation using FITC F4/80 and PE CD206. ^*^p<0.05, ^**^p<0.01, ^***^p<0.001. *ns* no statistical significance, *NC* negative control, *HG* high-glucose medium, *PUE* puerarin, *TNF-α* tumor necrosis factor-α, *IL*, interleukin

### Histopathological changes in the wound tissue of diabetic mice after puerarin treatment

As mentioned above, puerarin treatment can significantly promote the wound healing of diabetic mice. Thus, the relevant histopathological changes in wound tissues were further evaluated by H&E and MT staining on day 10. As we expected, the disease model group showed an epithelial abnormality and obvious structural alterations (Figure 2a). There was a large number of inflammatory infiltration cells, and collagen disorder and loss were also observed (Figure 2a). Puerarin treatment could significantly reverse this unfavorable trend (Figure 2a). The histological wound healing scores of the STZ + PUE group were also significantly higher than those of the STZ group (Figure 2b). After treatment with puerarin, the inflammation cells were reduced in the epithelium tissue of diabetic mice, and the amounts of Ly6G-labeled neutrophils and F4/80-branded macrophages were also decreased significantly (Figure 2c). Since STAT3 can regulate the biological behavior of immune cells through activation of extracellular signals, it is indispensable in the creation of chronic inflammation. Therefore, the expression of p-STAT3 in traumatic tissues was detected. As demonstrated in Figure 2c, the intensity of p-STAT3 in the STZ group was increased compared to that in the NC group, and puerarin treatment markedly downregulated the expression of p-STAT3 in the wound tissues of diabetic mice.

### Puerarin treatment attenuates inflammation in diabetic wounds

To further verify our experimental results, we performed immunofluorescence staining on both neutrophils and macrophages in wound tissue. The STZ group showed a remarkably higher infiltration of neutrophils and macrophages than the NC group, and the infiltration of these inflammatory cells was remarkably alleviated after puerarin treatment, which was comparable to that in the NC group (Figure 3a, b). At the same time, we also found that the formation of K5 was seriously attenuated in the STZ group and recovered after puerarin treatment (Figure 3a, b). It was found that the proportion of macrophages classified as M2 increased significantly in the STZ + PUE group (Figure 3c). On the other hand, M1 macrophages were markedly reduced in the STZ + PUE group (Figure 3d).

To determine the potential effect of puerarin on macrophage polarization, full-thickness skin defect wound margin tissues from mice were harvested for analysis on day 10 after modeling. RT-qPCR was conducted to analyze the expression of macrophage polarization-related cytokines. The findings demonstrated that F4/80, TNF-α, IL-1β and integrin alpha M (Cd11b) in the STZ group were upregulated compared with those in the NC group (Figure 4a–d). After treatment with puerarin, the expression levels of F4/80, TNF-α, IL-1β and Cd11b were remarkably downregulated, while those of Arg-1, IL-10 and TGF-β1 were significantly upregulated (Figure 4e–g). Inflammatory cytokines were also detected at the protein level, and the inflammatory protein expression decreased after puerarin treatment in diabetic mice (Figure 4h, i). These results indicate that puerarin has a significant therapeutic effect on the polarization of inflammatory infiltrating macrophages. Puerarin treatment can increase the levels of anti-inflammatory M2 macrophages. This also supports that puerarin has a negative role in regulating the pro-inflammatory response. As a whole, our findings indicate that puerarin can effectively improve inflammatory cell infiltration and M2 macrophage polarization during the repair of diabetic skin wounds.

### Puerarin inhibits NF-κB and MAPK signal transduction in the wound tissue of diabetic mice

MPO is produced by neutrophils and serves as a biomarker of inflammation. The activity of MPO was determined to quantify and reflect inflammatory cell infiltration in diabetic mice during wound healing. It was observed that wound healing induced excessive activation of MPO activity (Figure 5a). Interestingly, administration of puerarin markedly reversed these changes. As puerarin can negatively affect pro-inflammatory responses, the serum levels of inflammatory factors were further examined by ELISA. It was found that the serum levels of IL-6 and TNF-α increased substantially in the STZ group compared to the NC group (Figure 5b, c). However, the increased levels of IL-6 and TNF-α were inhibited by puerarin treatment. Notably, the mice in the STZ group exhibited a significant decrease in serum IL-10 levels, while puerarin could restore these levels (Figure 5d). To further clarify the potential mechanism, we have studied two important signaling cascades, namely, NF-κB and MAPK, which play a role in regulating diabetic wound healing. Through western blot analysis of wound tissue, we found that puerarin treatment significantly decreased the levels of p-IkBα, p-p65 and p65 (Figure 5e, f). In addition, consistent with the downregulation of the MAPK pathway, the STZ-induced phosphorylation levels of ERK, JNK and p38 were markedly increased in diabetic mice after puerarin treatment (Figure 5e, f). These results indicate that puerarin treatment inhibits p65, IkBα, ERK, JNK and p38. Moreover, the protein levels of M2 markers (i.e. Arg-1 and CD206) differed greatly on days 5 and 10 post-injury (Figure 5g, h). Therefore, it can be inferred that puerarin treatment is effective in suppressing the activation of inflammatory pathways and promoting macrophage M2 polarization during wound healing.

### Puerarin increases the proportion of M2 polarization *in vitro* via NF-κB and MAPK signaling cascades

RAW264.7 macrophages were cultured at various glucose concentrations and their survival rates were assessed by CCK8 assays. It was found that the survival rate of RAW264.7 cells decreased significantly when the glucose concentration was 50 mmol/l (Figure 6a). Next, cytotoxicity experiments for puerarin were performed and we found that puerarin had no toxic effect on macrophages (Figure 6a). To clarify the role of puerarin in inflammation, the macrophages were cultured in a high-glucose environment, and LPS (100 ng/ml) was added into the medium to create an inflammatory environment. The proteins involved in inflammation pathways (i.e. p65, p-IkBα and NF-κB/MAPK) in high glucose-induced RAW264.7 cells were evaluated by western blot assay. The findings demonstrated that p65, p-IkBα, p-JNK, p-p38 and p-ERK were upregulated in the high-glucose culture compared with the normal-glucose culture, and were downregulated after puerarin treatment (Figure 6b). There was no obvious difference in the expression of these proteins between the NC and PUE groups (Figure 6b). Additionally, RT-qPCR experiments were performed, and we found that TNF-α and IL-1β were significantly downregulated while IL-10, Arg-1, CD206 and CD163 were significantly upregulated after puerarin treatment (Figure 6c). These findings demonstrated that the proportion of M2 macrophages was elevated after puerarin treatment in the high-glucose environment. To further verify whether puerarin can promote macrophage M2 polarization in a high-glucose environment, flow cytometry and cellular immunofluorescence were performed. The results showed the same trend as previous experiments (Figure 6 d–g). In summary, puerarin can inhibit inflammation and promote M2 polarization of macrophages in a high-glucose environment via activation of the NF-κB/MAPK signaling axis.

## Discussion

The skin of diabetes patients is easily damaged and difficult to treat, which can affect their health status. Previous studies have shown that puerarin exhibits a protective impact on diabetic nephropathy and neuropathy through its anti-inflammatory ability [30]. Therefore, we investigated whether puerarin can enhance wound healing in diabetic mice. In this study, the following conclusions can be drawn: (1) intraperitoneal injection of puerarin can promote wound healing in diabetic mice; (2) puerarin can reduce wound inflammation and neutrophil infiltration by suppressing the activation of NF-κB and MAPK pathways in diabetic mice; and (3) puerarin regulates the polarization of M2 macrophages both *in vitro* and *in vivo* in a high-glucose environment. It is speculated the puerarin exerts an anti-inflammatory effect by promoting a rapid transition from the inflammatory stage to the remodeling stage. Our findings provide an important theoretical basis for treating refractory skin wound in diabetes patients.

Wound healing can be classified into four phases: hemostasis, inflammation, proliferation and remodeling. All processes are intertwined, and persistent inflammation can adversely affect subsequent tissue regeneration [31]. Hyperglycemia and vasculopathy caused by hyperglycemia around diabetic wounds can prolong inflammation and delay wound healing [32]. In our study, the wound healing of diabetic mice was considered to be slow. Delayed wound healing can lead to wound infection, destruction of the vascular bed, and then worsen the wound healing process, resulting in a vicious cycle [33]. In diabetes patients, it is crucial to stop this vicious circle as soon as possible. Previous research has shown that the wound healing process can be improved by inhibiting inflammatory responses in mice, which in turn accelerates the repair of inflammatory symptoms [34]. According to immunohistochemical staining and RT-qPCR, puerarin treatment reduces the infiltration of macrophages and neutrophils in diabetic wounds. This is beneficial for the wound healing process from the inflammatory period to the remodeling period.

The failure of chronic wound closure is also related to the disturbance of wound microenvironment that is characterized by increased levels of pro-inflammatory factors, mainly IL-1β, IL-6 and TNF-α [35]. These cytokines can modulate the polarization of macrophages, leading to improved wound healing [36]. The functions of IL-6 have received increasing attention, partly because of its anti-inflammatory and pro-inflammatory effects [23] and the role of IL-6 in mediating early injury responses *in vivo* via STAT3 [37]. Delayed wound healing is also related to the aggressive occurrence of an IL-1β pro-inflammatory response in diabetic mice [38]. In addition, previous studies have shown that these proinflammatory factors downregulate the wound healing effect mediated by TGF-β, which polarizes macrophages into a pro-inflammatory phenotype and impairs the wound healing response [39]. Therefore, the decreases in these proinflammatory factors can be considered as proof of the efficacy of puerarin treatment. Furthermore, when puerarin was applied to treat STZ-induced diabetic mice, we observed that IL-1β and TNF-α were remarkably downregulated at the transcriptional level, and p-STAT3 was decreased at the translational level, indicating that puerarin can effectively inhibit inflammatory factors during diabetic wound healing.

Macrophages are a heterogeneous cell population that can be activated by classical M1 or alternative M2 signals. Previous research has shown that M1 macrophages play a vital role in the early stage of wound healing by producing high levels of pro-inflammatory cytokines and promoting a persistent inflammatory response [40]. Polarization of M2 macrophages has been shown to be related to the acceleration of tissue repair processes. Growing evidence shows that M2 macrophages regulate collagen production, myofibroblast differentiation, fibroblast regeneration and re-angiogenesis during wound healing [41,42]. Several studies have also shown that a decrease in M2 macrophages can downregulate the levels of growth factors in the proliferation stage during wound healing [43,44]. In addition, increased levels of pro-inflammatory factors, such as iNOS and IL-1β, are associated with the non-healing phenotype [45]. Considering that M2 macrophages can promote wound repair, it is postulated that increasing the number of M2 macrophages in a wound may accelerate wound healing. In this study, we observed that TNF-α and IL-1β, which promote the polarization of M1 macrophages, were significantly reduced after treatment; while IL-10, Arg-1, CD206 and CD163, which induce M2 macrophage formation, were significantly increased. This indicates that puerarin treatment regulates M2 macrophages in the process of wound repair. During the process of wound healing, M2 macrophages dominate the wound healing site, with fewer M1 macrophages present after day 5. Most of the macrophages disappeared shortly after day 10 [46]. Furthermore, we were concerned that if we detected M2 macrophage indicators too early, puerarin might not have fully exerted its pharmacological role. Therefore, days 5 and 10 were selected to observe the indicators of macrophages.

Based on its role in upregulating the expression of proinflammatory genes, NF-κB/MAPK has been considered as a classical pro-inflammatory signaling axis. Under normal circumstances, the NF-κB and MAPK pathways are overactivated in the process of diabetic skin wound repair. Moreover, these two pathways are closely related to macrophage polarization [47]. This study also found that STZ-induced diabetic mouse trauma could significantly activate NF-κB and MAPK signaling cascades. As a common transcription factor, NF-κB is involved in the transcription of various genes related to inflammation and the immune response. Recent studies have confirmed that puerarin can reduce acute inflammatory injury through restraining the Toll-like receptor 4 (TLR4)/Myeloid differentiation primary response gene 88 (MyD88)/NF-κB signaling axis [48]. Other scholars have shown that puerarin can reduce the release of inflammatory mediators by inhibiting the activation of TLR4, p38 MAPK and ERK1/2 [49]. Our western blot results also indicated that puerarin treatment could inhibit the NF-κB/MAPK signaling axis in a high-glucose environment. It has been reported that inhibition of NF-κB and MAPK signaling pathways could enhance macrophage differentiation into the proinflammatory M2 phenotype [50,51]. According to our results, puerarin inhibited NF-κB and MAPK signaling pathways, thereby playing an anti-inflammatory role in diabetic wound healing. Meanwhile, our study demonstrated that puerarin increased expression of IL-10 and Arg-1, which was important for inducing the polarization of M2 macrophages. Conversely, the M1 macrophage-induced levels of TNF-α and IL-1β were downregulated. Thus, macrophage M2 polarization and inhibition of the NF-κB/MAPK pathway play a synergistic role in ameliorating diabetic wounds. Nevertheless, the mechanisms underlying the anti-inflammatory effect of puerarin on diabetic wound healing need to be explored in future research.

## Conclusions

In summary, our findings demonstrate that the traditional Chinese medicine extract puerarin has a significant therapeutic effect on wound healing in diabetic mice, and this study proves that such therapeutic effect is achieved by restraining the activation of inflammatory pathways and modulating macrophage polarization. Therefore, puerarin is potentially effective in treating diabetic wounds.

## Abbreviations

Arg-1: Arginase 1; BG: Blood glucose; Cd11b: Integrin alpha M; CD206: Cluster of differentiation 206; DAPI: 4′,6-Diamidino-2-phenylindole; F4/80: Mouse EGF-like module-containing mucin-like hormone receptor-like 1; GAPDH: Glyceraldehyde 3-phosphate dehydrogenase; H&E: Hematoxylin and eosin staining; HG: High-glucose medium; IL-1β: Interleukin-1β; iNOS: Inducible nitric oxide synthase; K5: Cytokeratin 5; Ly6G: Lymphocyte antigen 6 complex; MT: Masson’s trichrome; NF-κB: Nuclear factor kappa B; NC: Negative control; PUE: Puerarin; STATs: Signal transducers and activators of transcription; STZ: Streptozotocin; p-ERK: Phospho-extracellular signal-related kinase; p-IkBα: Phospho-nuclear factor of kappa alpha; p-JNK: Phospho-c-Jun N-terminal kinase; p-STAT3: Phosphorylated signal transducer and activator of transcription-3; p65: Nuclear factor kappa B p65; p-p38: Phospho-p38 MAP kinase; TNF-α: Tumor necrosis factor-α; PI3K: Phosphatidylinositol 3-kinase; AKT: Protein kinase B; ROS: Reactive oxygenspecies; DMEM: Dulbecco's modified Eagle's medium; FBS: Foetal bovine serum; CCK8: Cell counting kit-8; BSA: Bovine serum albumin; ELISA: enzyme-linkedimmunosorbent assay; FITC: Fluorescein isothiocyanate; PE: P-phycoerythrin;ANOVA: Analysis of variance; TLR4: Toll-like receptor 4; MyD88: Myeloiddifferentiation primary response gene 88; i.p.: intraperitoneal injection; RT-qPCR:Real time quantitative polymerase chain reaction; SDS-PAGE: Sodium dodecylsulfate polyacrylamide gel electrophoresis; PVDF: Polyvinylidene fluoride; TBST: Tris buffered saline tween; HRP: Horse radish peroxidase; PBST: Phosphate-bufferedsaline tween.

## Data Availability

The datasets used are available from the corresponding author on reasonable request.

## References

[1] Rodrigues M , Kosaric N, Bonham CA, Gurtner GC. Wound healing: a cellular perspective. Physiol Rev. 2019;99:665–706. 30475656 10.1152/physrev.00067.2017 PMC6442927

[2] Ren H , Zhao F, Zhang Q, Huang X, Wang Z. Autophagy and skin wound healing. *Burns Trauma*. 2022;10:tkac003. 10.1093/burnst/tkac003. PMC8847901 35187180

[3] Diegelmann RF , Evans MC. Wound healing: an overview of acute, fibrotic and delayed healing. Front Biosci. 2004;9:283–9. 14766366 10.2741/1184

[4] Han CM , Cheng B, Wu P. Clinical guideline on topical growth factors for skin wounds. *Burns Trauma*. 2020;8:tkaa035. 10.1093/burnst/tkaa035. PMC7520573 33015207

[5] Ridiandries A , Tan J, Bursill CA. The role of chemokines in wound healing. Int J Mol Sci. 2018;19(10):3217. 10.3390/ijms19103217. PMC6214117 30340330

[6] Liu W , Yu M, Xie D, Wang L, Ye C, Zhu Q, et al. Melatonin-stimulated MSC-derived exosomes improve diabetic wound healing through regulating macrophage M1 and M2 polarization by targeting the PTEN/AKT pathway. Stem Cell Res Ther. 2020;11:259. 10.1186/s13287-020-01756-x. 32600435 PMC7322868

[7] Tu ZL , Lin C. Research advances on the effects and mechanism of curcumin in promoting diabetic wound healing. CHINESE JOURNAL OF BURNS AND WOUNDS. 2021;37:391–4. 10.3760/cma.j.cn501120-20200224-00089 PMC11917206 33887887

[8] Hesketh M , Sahin KB, West ZE, Murray RZ. Macrophage phenotypes regulate scar formation and chronic wound healing. Int J Mol Sci. 2017;18(7):1545. 10.3390/ijms18071545. PMC5536033 28714933

[9] Kimball AS , Davis FM, DenDekker A, Joshi AD, Schaller MA, Bermick J, et al. The histone methyltransferase Setdb2 modulates macrophage phenotype and uric acid production in diabetic wound repair. Immunity. 2019;51:258–271.e5. 31350176 10.1016/j.immuni.2019.06.015 PMC6703945

[10] Gallagher KA , Joshi A, Carson WF, Schaller M, Allen R, Mukerjee S, et al. Epigenetic changes in bone marrow progenitor cells influence the inflammatory phenotype and alter wound healing in type 2 diabetes. Diabetes. 2015;64:1420–30. 25368099 10.2337/db14-0872 PMC4375075

[11] Martinez FO , Sica A, Mantovani A, Locati M. Macrophage activation and polarization. Front Biosci. 2008;13:453–61. 17981560 10.2741/2692

[12] Feng J , Dong C, Long Y, Mai L, Ren M, Li L, et al. Elevated Kallikrein-binding protein in diabetes impairs wound healing through inducing macrophage M1 polarization. Cell Commun Signal. 2019;17:60. 10.1186/s12964-019-0376-9. 31182110 PMC6558923

[13] Verreck FA , de Boer T, Langenberg DM, Hoeve MA, Kramer M, Vaisberg E, et al. Human IL-23-producing type 1 macrophages promote but IL-10-producing type 2 macrophages subvert immunity to (myco)bacteria. Proc Natl Acad Sci U S A. 2004;101:4560–5. 15070757 10.1073/pnas.0400983101 PMC384786

[14] Gordon S , Martinez FO. Alternative activation of macrophages: mechanism and functions. Immunity. 2010;32:593–604. 20510870 10.1016/j.immuni.2010.05.007

[15] Liu YC , Zou XB, Chai YF, Yao YM. Macrophage polarization in inflammatory diseases. Int J Biol Sci. 2014;10:520–9. 24910531 10.7150/ijbs.8879 PMC4046879

[16] Orecchioni M , Ghosheh Y, Pramod AB, Ley K. Macrophage polarization: different gene signatures in M1(LPS+) vs. classically and M2(LPS-) vs. Alternatively Activated Macrophages Front Immunol. 2019;10:1084. 10.3389/fimmu.2019.01084. 31178859 PMC6543837

[17] Nelson DE , Ihekwaba AE, Elliott M, Johnson JR, Gibney CA, Foreman BE, et al. Oscillations in NF-kappaB signaling control the dynamics of gene expression. Science. 2004;306:704–8. 15499023 10.1126/science.1099962

[18] Lawrence T . The nuclear factor NF-kappaB pathway in inflammation. Cold Spring Harb Perspect Biol. 2009;1:a001651. 10.1101/cshperspect.a001651. 20457564 PMC2882124

[19] Wu R , Chen F, Wang N, Tang D, Kang R. ACOD1 in immunometabolism and disease. Cell Mol Immunol. 2020;17:822–33. 32601305 10.1038/s41423-020-0489-5 PMC7395145

[20] Ganesh GV , Ramkumar KM. Macrophage mediation in normal and diabetic wound healing responses. Inflamm Res. 2020;69:347–63. 32146517 10.1007/s00011-020-01328-y

[21] Yu T , Gan S, Zhu Q, Dai D, Li N, Wang H, et al. Modulation of M2 macrophage polarization by the crosstalk between Stat6 and Trim24. Nat Commun. 2019;10:4353. 10.1038/s41467-019-12384-2. 31554795 PMC6761150

[22] Horwood NJ . Macrophage polarization and bone formation: a review. Clin Rev Allergy Immunol. 2016;51:79–86. 26498771 10.1007/s12016-015-8519-2

[23] Fernando MR , Reyes JL, Iannuzzi J, Leung G, McKay DM. The pro-inflammatory cytokine, interleukin-6, enhances the polarization of alternatively activated macrophages. PLoS One. 2014;9:e94188. 10.1371/journal.pone.0094188. 24736635 PMC3988054

[24] McFarland BC , Gray GK, Nozell SE, Hong SW, Benveniste EN. Activation of the NF-kappaB pathway by the STAT3 inhibitor JSI-124 in human glioblastoma cells. Mol Cancer Res. 2013;11:494–505. 23386688 10.1158/1541-7786.MCR-12-0528 PMC3656973

[25] Zhou YX , Zhang H, Peng C. Puerarin: a review of pharmacological effects. Phytother Res. 2014;28:961–75. 24339367 10.1002/ptr.5083

[26] Chen X , Qian L, Wang B, Zhang Z, Liu H, Zhang Y, et al. Synergistic Hypoglycemic effects of pumpkin polysaccharides and Puerarin on type II diabetes mellitus mice. Molecules. 2019;24:955. 10.3390/molecules24050955. PMC6429091 30857163

[27] Chen X , Yu J, Shi J. Management of Diabetes Mellitus with Puerarin, a natural Isoflavone from Pueraria lobata. Am J Chin Med. 2018;46:1771–89. 30525896 10.1142/S0192415X18500891

[28] Bai YL , Han LL, Qian JH, Wang HZ. Molecular mechanism of Puerarin against diabetes and its complications. Front Pharmacol. 2022;12:780419. 10.3389/fphar.2021.780419. PMC8764238 35058775

[29] van de Vyver M , Boodhoo K, Frazier T, Hamel K, Kopcewicz M, Levi B, et al. Histology scoring system for murine cutaneous wounds. Stem Cells Dev. 2021;30:1141–52. 34130483 10.1089/scd.2021.0124 PMC9022171

[30] Wu Y , Xue B, Li X, Liu H. Puerarin prevents high glucose-induced apoptosis of Schwann cells by inhibiting oxidative stress. Neural Regen Res. 2012;7:2583–91. 25368634 10.3969/j.issn.1673-5374.2012.33.003 PMC4200725

[31] Martin P , Nunan R. Cellular and molecular mechanisms of repair in acute and chronic wound healing. Br J Dermatol. 2015;173:370–8. 26175283 10.1111/bjd.13954 PMC4671308

[32] Morton LM , Phillips TJ. Wound healing and treating wounds: differential diagnosis and evaluation of chronic wounds. J Am Acad Dermatol. 2016;74:589–605 quiz 605-6. 26979352 10.1016/j.jaad.2015.08.068

[33] Ricco JB , Thanh PL, Schneider F, Illuminati G, Belmonte R, Valagier A, et al. The diabetic foot: a review. J Cardiovasc Surg. 2013;54:755–62. 24126512

[34] Davis FM , Kimball A, Boniakowski A, Gallagher K. Dysfunctional wound healing in diabetic foot ulcers: new crossroads. Curr Diab Rep. 2018;18:2. 10.1007/s11892-018-0970-z. 29362914

[35] Werner S , Grose R. Regulation of wound healing by growth factors and cytokines. Physiol Rev. 2003;83:835–70. 12843410 10.1152/physrev.2003.83.3.835

[36] Kim SY , Nair MG. Macrophages in wound healing: activation and plasticity. Immunol Cell Biol. 2019;97:258–67. 30746824 10.1111/imcb.12236 PMC6426672

[37] Lin ZQ , Kondo T, Ishida Y, Takayasu T, Mukaida N. Essential involvement of IL-6 in the skin wound-healing process as evidenced by delayed wound healing in IL-6-deficient mice. J Leukoc Biol. 2003;73:713–21. 12773503 10.1189/jlb.0802397

[38] Mirza RE , Fang MM, Ennis WJ, Koh TJ. Blocking interleukin-1beta induces a healing-associated wound macrophage phenotype and improves healing in type 2 diabetes. Diabetes. 2013;62:2579–87. 23493576 10.2337/db12-1450 PMC3712034

[39] Laato M , Heino J, Gerdin B, Kahari VM, Niinikoski J. Interferon-gamma-induced inhibition of wound healing in vivo and in vitro. Ann Chir Gynaecol Suppl. 2001;215:19–23. 12016743

[40] Qing L , Fu J, Wu P, Zhou Z, Yu F, Tang J. Metformin induces the M2 macrophage polarization to accelerate the wound healing via regulating AMPK/mTOR/NLRP3 inflammasome singling pathway. Am J Transl Res. 2019;11:655–68. 30899369 PMC6413292

[41] Wynn TA , Vannella KM. Macrophages in tissue repair, regeneration, and fibrosis. Immunity. 2016;44:450–62. 26982353 10.1016/j.immuni.2016.02.015 PMC4794754

[42] Snyder RJ , Lantis J, Kirsner RS, Shah V, Molyneaux M, Carter MJ. Macrophages: a review of their role in wound healing and their therapeutic use. Wound Repair Regen. 2016;24:613–29. 27196106 10.1111/wrr.12444

[43] Sica A , Erreni M, Allavena P, Porta C. Macrophage polarization in pathology. Cell Mol Life Sci. 2015;72:4111–26. 26210152 10.1007/s00018-015-1995-y PMC11113543

[44] Okizaki S , Ito Y, Hosono K, Oba K, Ohkubo H, Amano H, et al. Suppressed recruitment of alternatively activated macrophages reduces TGF-beta1 and impairs wound healing in streptozotocin-induced diabetic mice. Biomed Pharmacother. 2015;70:317–25. 25677561 10.1016/j.biopha.2014.10.020

[45] Mirza RE , Fang MM, Novak ML, Urao N, Sui A, Ennis WJ, et al. Macrophage PPARgamma and impaired wound healing in type 2 diabetes. J Pathol. 2015;236:433–44. 25875529 10.1002/path.4548 PMC4509817

[46] Louiselle AE , Niemiec SM, Zgheib C, Liechty KW. Macrophage polarization and diabetic wound healing. Transl Res. 2021;236:109–16. 34089902 10.1016/j.trsl.2021.05.006

[47] Porta C , Riboldi E, Ippolito A, Sica A. Molecular and epigenetic basis of macrophage polarized activation. Semin Immunol. 2015;27:237–48. 26561250 10.1016/j.smim.2015.10.003

[48] Xu Y , Xiong Y, Xu C, Xu C. Standard Puerarin prevents diabetic renal damage by inhibiting miRNA-140-5p expression. Diabetes Metab Syndr Obes. 2020;13:3947–58. 33122931 10.2147/DMSO.S273952 PMC7591269

[49] Deng H , Wang X, Sun H, Xiao X. Puerarin inhibits expression of tissue factor induced by oxidative low-density lipoprotein through activating the PI3K/Akt/eNOS pathway and inhibiting activation of ERK1/2 and NF-κB. Life Sci. 2017;191:115–21. 29037842 10.1016/j.lfs.2017.10.018

[50] He S , Hu Q, Xu X, Niu Y, Chen Y, Lu Y, et al. Advanced glycation end products enhance M1 macrophage polarization by activating the MAPK pathway. Biochem Biophys Res Commun. 2020;525:334–40. 32093892 10.1016/j.bbrc.2020.02.053

[51] Xu Y , Liao C, Liu R, Liu J, Chen Z, Zhao H, et al. IRGM promotes glioma M2 macrophage polarization through p62/TRAF6/NF-kappaB pathway mediated IL-8 production. Cell Biol Int. 2019;43:125–35. 30288851 10.1002/cbin.11061 PMC13397409

